# Improved Performances of Zn//MnO_2_ Batteries with an Electrolyte Containing Co-Additives of Polyethylene Glycol and Lignin Derivatives

**DOI:** 10.3390/polym17070888

**Published:** 2025-03-26

**Authors:** Muzammil Hussain Memon, Md. Asraful Alam, Qiyuan Xie, Abdul Rahman Abbasi, Lele Wang, Jingliang Xu, Wenlong Xiong

**Affiliations:** 1 State Key Laboratory of Cotton Bio-breeding and Integrated Utilization, School of Chemical Engineering, Zhengzhou University, Zhengzhou 450001, China; muzhusain.mh@outlook.com (M.H.M.); alam@zzu.edu.cn (M.A.A.); xieqiyuan@gs.zzu.edu.cn (Q.X.); rahmanabbasi56@yahoo.com (A.R.A.); wll2024@gs.zzu.edu.cn (L.W.); 2 State Key Laboratory of Bio-based Transportation Fuel Technology, School of Chemical Engineering, Zhengzhou University, Zhengzhou 450001, China; 3 Henan Center for Outstanding Overseas Scientists, Zhengzhou University, Zhengzhou 450001, China

**Keywords:** polyethylene glycol, kraft lignin, zinc-ion batteries, electrolyte additives, quaternization

## Abstract

Multi-component electrolyte additives may significantly contribute to improving the performance of rechargeable aqueous zinc-ion batteries. Herein, we propose a mixed electrolyte system employing polyethylene glycol 200 (PEG200) and quaternized kraft lignin (QKL) as co-additives in Zn//MnO_2_ batteries. Reduced corrosion and the suppression of the hydrogen evolution reaction on the zinc electrode were achieved when 0.5 wt.% of PEG200 and 0.2 wt.% of QKL were added to the reference aqueous electrolyte. This optimized electrolyte, 0.5% PEG200 + 0.2% QKL, was conducive to improving Zn reversibility in Zn//Zn symmetric batteries and resulted in higher cycling stability, with a coulombic efficiency of 98.01% under 1 mA cm^−2^ and 1 mAh cm^−2^ for Zn//Cu cells. Furthermore, Zn//MnO_2_ full batteries with 0.5% PEG200 + 0.2% QKL presented good overall electrochemical performance and exhibited a decent discharge capacity of around 85 mAh g^−1^ after 2000 cycles at 1.5 A g^−1^. As confirmed by X-ray diffraction and scanning electron microscopy, a dominant (002) oriental dendrite-free Zn deposition was achieved on the zinc anode of the battery using 0.5% PEG200 + 0.2% QKL, and the byproducts were also reduced significantly. This study has contributed to the development of electrolyte co-additives for zinc-ion batteries.

## 1. Introduction

The emergence of various new types of secondary batteries has paved the way for promising advancements in energy storage technologies [1,2,3]. Zinc-ion batteries (ZIBs) have shown significant potential to be utilized in large-scale energy storage systems because of the higher volumetric energy density (5855 mAh cm^−3^), low redox potential (−0.76 V) versus standard hydrogen electrodes, and high safety associated with aqueous electrolytes [3,4,5]. In addition, the richness of Zn reserves, as well as low toxicity and costs, are also prominent advantages [3,4,5]. However, the thermodynamic instability of the Zn metal in aqueous environments is of the utmost concern, as it impedes prolonged reversibility and thus hinders practical applications [3,4,5]. Corrosion and dendritic growth hinder the lifespan of a battery, as these undesirable phenomena deteriorate the Zn anode in a water-based electrolyte. Furthermore, because of the elevated OH^−^ concentration induced by H_2_ evolution, the local alkalinity could also rise [6,7]. This would quickly result in the formation of inactive byproducts like Zn(OH)_2_ or Zn_4_SO_4_(OH)_6_⋅xH_2_O, which would randomly passivate the fresh Zn surface [8]. Subsequently, the low coulombic efficiency (CE) attributed to the hydrogen evolution reaction (HER) and interrelated corrosion further deteriorate the Zn anode by forming more dendrites and byproducts and ultimately consuming the negative electrode, with the result of internal short-circuiting [9,10].

To protect the Zn anode in an aqueous electrolyte, artificial interphase layers [11], a 3D structural design [12], the adoption of Zn alloys [13], and electrolyte optimization [11,14] have all been demonstrated to reduce water-side reactions and dendrite formation on the electrode. Recently, the introduction of additives into electrolytes has emerged as an effective and facile strategy. The role of additives is mainly to inhibit Zn anode problems such as corrosion, HER, and uncontrolled dendritic growth. Due to the presence of numerous functional groups, electrolyte additives can regulate the solvation structure and guide Zn^2+^ flux, which have significant effects in terms of reducing water-related side reactions and dendrite formation on the Zn anode [14,15,16,17,18]. Organic additives are mainly reported for ZIBs due to the variety of functional groups they contain, including hydroxyl, carbonyl, and ammonium groups, with the ability to control uniform Zn deposition. Such functional groups possess the ability to adsorb onto the Zn anode, forming an electrostatic-shielding layer that limits dendrites and supports homogeneous Zn deposition [19,20].

Polyethylene glycol (PEG) with abundant hydroxyl functional groups has the tendency to adsorb onto the Zn anode and guide Zn ion flux to distribute homogeneously, thus restricting dendrites. Although PEG dominates the 002-plane of Zn deposition, the molecules are not fully reversible and thus block the adsorption, sites implying reduced capacity during cycling [21,22,23,24]. Therefore, PEG in high dosages can introduce several challenges that may negatively affect the rate performance due to increased viscosity, which can raise the cost of ZIBs. Currently, using low-cost and biodegradable lignin derivatives as electrolyte additives has exhibited potential in the context of ZIBs [25]. In particular, cationic lignin has the capacity to form an electrostatic shielding layer and control the solvation structure of zinc ions that influence the nucleation and de-solvation of hydrated Zn^2+^ ions on the metallic Zn surface, resulting in the consistently uniform deposition of a Zn layer [26,27]. Although adding single-component electrolyte additives has been widely studied, it is still necessary to consider adding multi-component electrolyte additives due to the relatively limited function of a single electrolyte additive [28,29]. The development of multi-component electrolyte additives that are nontoxic and inexpensive and require only low dosages is still encountering enormous challenges. PEG200 has a relatively low molecular weight, which results in lower viscosity as compared to other PEGs with a higher molecular weight [30]. The other polymers, such as polyethylene oxide, polyvinyl alcohol, and polyacrylonitrile, possess a restricted ability to conduct ions [31,32], while it was shown that quaternized kraft lignin (QKL) basically does not reduce the ionic conductivity of the electrolyte [33]. In this regard, PEG200 and QKL may play synergistic roles in protecting the zinc anode when used as electrolyte co-additives for ZIBs.

Herein, we present a combined additive strategy using PEG200 and QKL. QKL is synthesized via the quaternization of green and renewable kraft lignin, which is the most abundant industrial lignin produced by the pulp and paper industry [34,35,36]. The quaternary ammonium groups, (CH_3_)_3_N^+^, grafted on lignin enhance the solubility and dispersion of QKL in an aqueous environment. The addition of QKL with PEG200 as co-additives can improve the Zn nucleation process, which helps to improve discharge capacity during cycling. Due to the presence of positively charged groups, (CH_3_)_3_N^+^, QKL demonstrates a strong adsorption ability and is able to form an electrostatic shielding layer on the Zn anode, which can facilitate the de-solvation process of Zn^2+^ ions and increase Zn nucleation sites. As shown in the Fourier transform infrared spectroscopy (FTIR) and Raman spectra results, QKL also has an effect on the Zn^2+^ coordination environment, which can reduce water-side reactions. Meanwhile, PEG200 supports (002) textured Zn deposition and thus restricts dendritic development during the Zn stripping/plating process. Subsequently, Zn//Zn symmetric batteries exhibit improved cycling stability for 500 h at 0.5 mA cm^−2^ and around 280 h at 1 mA cm^−2^. For Zn//MnO_2_ full cells, 0.5% PEG200 + 0.2% QKL is able to retain a higher discharge capacity as compared to REF and can achieve long-term cycling stability for 2000 cycles, with decent capacity retention.

## 2. Materials and Methods

### 2.1. Materials

α-MnO_2_ (99.95%) and manganese sulfate monohydrate (MnSO_4_·1H_2_O, 99%) were purchased from Aladdin Co., Ltd. PEG200 was received from Macklin (Shanghai, China), whereas zinc sulfate heptahydrate (ZnSO_4_·7H_2_O, 98%) was obtained from Alfa Aesar. Kraft lignin (KL) was provided by Tiger Forest & Paper Group Co., Ltd., located in Changsha, China, and was purified via an acid precipitation method. Acetylene black, zinc foil (99.995% purity; thickness = 0.1 mm), conductive polyethylene (PE) film, polyvinylidene fluoride (PVDF, HSV 900, Arkema), and N-methyl-2-pyrrolidone (NMP) were received from Shenzhen Kejing Star Technology Company (Shenzhen, China). The glass fiber (GF) used as a separator was obtained from Zhejiang Shangde Energy Technology Co., Ltd. (Lishui, China). 3-chloro-2-hydroxypropyltrimethylammonium chloride (CHPTMAC) solution (60 wt.%) was purchased from Sigma-Aldrich (Shanghai, China), while sulfuric acid (H_2_SO_4_, 95–98 wt.%) and sodium hydroxide (NaOH, 96 wt.%) were obtained from Luoyang Chemical Reagent Factory (Luoyang, China).

### 2.2. Synthesis of Quaternized Kraft Lignin (QKL)

QKL was synthesized using the method presented in the published literature [26,33]. A three-necked flask with a capacity of 250 mL was filled with 100 g of kraft lignin (KL) solution, which contained a solid content of 25%. The KL solution was subjected to heating in a water bath with mechanical stirring until it reached 85 °C. Subsequently, a peristaltic pump was used to gradually add 26.66 g of CHPTMAC solution. The reaction proceeded for 5 h after the CHPTMAC solution was completely added. At the same time as the addition of the CHPTMAC solution, a suitable amount of NaOH solution (50 wt.%) was incrementally introduced into the reaction mixture in order to maintain the pH level above 11. After following purification techniques including dialysis, rotary evaporation, and vacuum freeze-drying, the final product, referred to as QKL, was obtained.

### 2.3. Fabrication of Batteries

Preparation of electrolyte: Here, 2 M ZnSO_4_ + 0.2 M MnSO_4_ aqueous solution was utilized as the reference electrolyte (REF), which was obtained by mixing ZnSO_4_·7H_2_O and MnSO_4_·1H_2_O. The pH of the electrolyte was adjusted to 4.5 ± 0.05 using sulfuric acid with 20% aqueous solution.

Preparation of electrolyte additives: The different dosages of additives by weight percent (wt.%) were added into REF to prepare electrolyte additives. Then, 0.5% PEG200 + 0.2% QKL was prepared with the addition of 0.1 g of PEG200, 0.04 g of QKL, and 19.86 g of REF. Similarly, 0.5% PEG200, 0.5% PEG200 + 0.1% QKL, and 0.5% PEG200 + 0.5% QKL were prepared accordingly.

Preparation of cathode: The working cathode was prepared with the composition of α-MnO_2_, with acetylene black as the conductive additive and PVDF suspended in NMP as a binder in the ratio of 70%, 20%, and 10%. The as-prepared slurry cast on PE film was dried under vacuum at 60 °C in an oven and cut into circular discs 12 mm in diameter for the coin cells’ assembly.

Preparation of Zn anode: After being polished for roughly 20 min with polishing powder, the pure zinc foil (0.1 mm thick) was cleaned with DI water and absolute ethyl alcohol. The polished zinc foil was cut into 12 mm diameter anode discs, which were utilized in the assembled batteries.

Assembly of batteries: The electrochemical performance was evaluated using type-2025 coin cells. The Zn//MnO_2_ full battery was composed of an α-MnO_2_ cathode, polished circular zinc (Zn) as the Zn anode, and glass fiber (GF) as the separator soaked in aqueous electrolytes (120 µL), as depicted in Figure S1a (Supporting Information). The symmetric cells were composed of Zn anodes as the cathode and anode, as shown in Figure S1b (Supporting Information).

### 2.4. Electrochemical Tests

The Zn//MnO_2_ full cells’ and Zn//Zn symmetric cells’ performances were assessed using a NEWARE battery tester (Neware Co. Ltd., Shenzhen, China) at room temperature. The cycling performance for Zn//MnO_2_ was assessed at 1.5 A g^−1^ after activation for 10 cycles at 0.1 A g^−1^, while rate performance was measured from 0.1 to 5 A g^−1^. The charge and discharge cut-off voltage for Zn//MnO_2_ were 1.9 and 1.0 V, respectively. The open circuit voltage (OCV) was tested to evaluate the self-discharge of full batteries under the rest condition for 24 h after being fully charged to 1.9 V at 0.1 A g^−1^. The CE test was performed on Zn//Cu asymmetric cells under 1 mA cm^−2^ and 1 mAh cm^−2^ with/without additives.

Chronoamperometry (CA), linear sweep voltammetry (LSV), electrochemical impedance spectroscopy (EIS), nucleation overpotential (NOP), and Tafel plot tests were conducted on CHI-604E, while the cyclic voltammetry (CV) of Zn//MnO_2_ coin cells at a scan rate of 0.1 mV s^−1^ was assessed on CHI-1040C electrochemical workstations. The CA and Tafel curves were measured using a three-electrode system with a Zn plate as the working electrode, platinum foil as the counter electrode, and Ag/AgCl as the reference electrode. The LSV and NOP tests were conducted on Zn//Ti half cells at a scan rate of 1 mV s^−1^, whereas EIS was performed on Zn//MnO_2_ cells from 0.1 to 10^5^ Hz, with an amplitude of 5 mV.

### 2.5. Material Characterization

The structural analyses of KL and QKL were characterized by FTIR, ^1^H NMR, Zeta potential, and elemental analysis. The electrolyte structure was characterized by FTIR (Brüker, Tensor II, Billerica, MA, USA) in attenuated total reflection mode (ATR), and Raman spectra were assessed with a 532 nm laser using Horriba, France, LabRAM HR Evo. The crystal structure and morphology of the Zn electrode were examined through X-ray diffraction (XRD) and scanning electron microscopy (SEM), respectively. Energy-dispersive spectroscopy (EDS) was performed on the Zn anode to determine zinc (Zn), sulfur (S), and oxygen (O) contents.

## 3. Results and Discussion

### 3.1. FTIR, ^1^H NMR, Zeta Potential, and Elemental Analysis for KL and QKL

**Figure 1:**
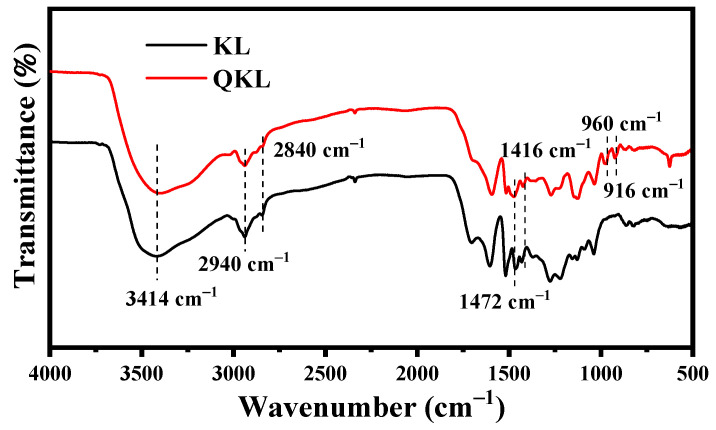
FTIR for KL and QKL.

To analyze the success of quaternization, FTIR, ^1^H NMR, Zeta potential, and elemental analysis characterization were performed on KL and QKL. As presented in Figure 1, the FTIR spectra test for KL and QL was performed to analyze the changes in the functional groups after the quaternization process. In the broad absorption spectrum ranging from 3600 to 3000 cm^−1^, both KL and QKL exhibited comparable bands corresponding to the presence of hydroxyl groups in their aromatic and aliphatic structures. The peak absorption at 2940 cm^−1^ primarily indicates C–H stretching linked to the methyl and methylene groups of their side chains, whereas the bands observed at 2840 cm^−1^ may have arisen due to the C–H stretching associated with aromatic methoxyl groups [37]. The peaks that appeared at 1591 and 1510 cm^–1^ can be attributed to the aromatic skeletal structure of KL. Compared to unmodified lignin, new absorption peaks were observed for QKL at 1416 and 960 cm^−1^, which correspond to C−N and the methyl group of the (–N^+^(CH_3_)_3_) [38]. In addition, the ^1^H NMR revealed the proton signal of the phenolic hydroxyl group for KL between 8.5 and 9.2 ppm, as shown in Figure S2a (Supporting Information). However, the proton signal disappeared for QKL, as displayed in Figure S2b (Supporting Information), indicating that the reaction site for the grafting of the quaternary ammonium group is the phenolic hydroxyl group [26,33]. Moreover, Table S1 (Supporting Information) presents the Zeta potential values for KL and QKL at pH 4.5. The average Zeta potential for KL was measured at −36.709 mV (negative) for KL, whereas QKL showed 23.3 mV (positive). The negative value of KL was neutralized in QKL, which can be attributed to the involvement of positively charged quaternary ammonium groups. The elemental analysis further confirmed the successful preparation of QKL with an increase in nitrogen content, as shown in Table S2 (Supporting Information), implying the successful grafting of quaternary ammonium groups (CH_3_)_3_N^+^ [26].

### 3.2. Changes in Zn^2+^ Solvation Structure

First, electrolyte additives were prepared by adding QKL and PEG200 into the aqueous electrolyte. The ionic conductivities were examined for all electrolytes with/without additives using a conductive probe, as presented in Table S3 (Supporting Information). As compared to REF, all additives demonstrated negligible reductions in ionic conductivity that were maintained around 39–43 mS cm^−1^, which is consistent with the values for electrolytes containing dual additives [27,39]. As shown, there was an uncompromising effect on the conductivity of ionic transport in aqueous electrolytes with co-additives.

**Figure 2:**
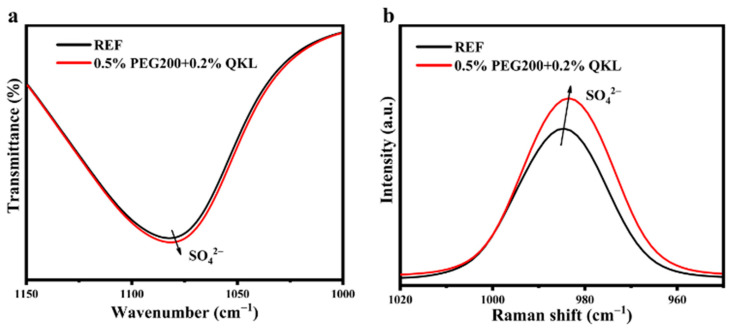
(**a**) FTIR and (**b**) Raman spectra for v-SO_4_^2−^ in REF and 0.5% PEG200 + 0.2% QKL.

Afterward, the as-prepared electrolyte additives were analyzed by FTIR to evaluate solvation structural changes. As revealed by FTIR in Figure S3a (Supporting Information), the peaks that correspond to O−H stretching, O−H bending, and SO_4_^2−^ vibrations were observed at 2900–3700 cm^−1^, 1600–1650 cm^−1^, and 1000–1150 cm^−1^, respectively, for the electrolytes with/without additives. However, with the addition of 0.5% of PEG200 and 0.2% of QKL into the electrolyte as a co-additive, the SO_4_^2−^ peak between 1150 and 1000 cm^−1^ shifted towards lower wavenumbers, as exhibited in Figure 2a, which indicates a reduction in electrostatic attraction between Zn^2+^ and SO_4_^2−^ [40]. The Raman spectra further confirmed a similar trend in the shifting of the SO_4_^2−^ group, as shown in Figure 2b, implying an alteration of Zn^2+^’s solvation structure. This weakening phenomenon can be attributed to the cationic quaternary ammonium group (CH_3_)_3_N^+^ of QKL, which is able to attract SO_4_^2−^ electrostatically [41]. Furthermore, the positively charged group of QKL can repel Zn^2+^ ions and thus suppress the formation of the side product Zn_4_SO_4_(OH)_6_·xH_2_O (ZHS). Meanwhile, 0.5% PEG200, as a singular additive, showed no shifting when added into the ZnSO_4_-based electrolyte as compared to co-additives after mixing with QKL, as confirmed by FTIR in Figure S3b (Supporting information), thus implying that QKL is mainly responsible for changes in the solvation structure of Zn^2+^ ions. Concomitantly, the role of PEG200 typically involves the guidance of Zn ion flux across the negative anode’s surface, with preferential adsorption.

### 3.3. Zn Deposition Behavior in REF and 0.5% PEG200 + 0.2% QKL

**Figure 3:**
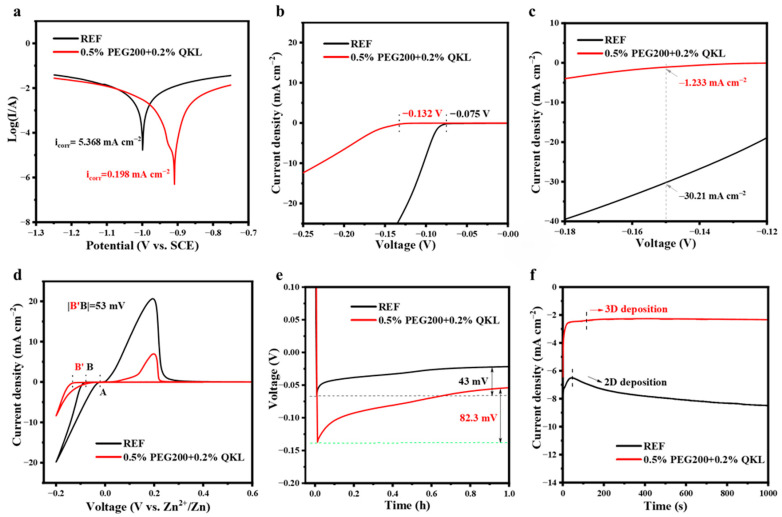
Mechanism of enhanced cycling stability in 0.5% PEG200 + 0.2% QKL. (**a**) Corrosion rate (Tafel plot method) of the Zn anode tested at a 0.1 mV s^−1^ scan rate. (**b**) LSV of Zn//Ti cells at 1 mV s^−1^. (**c**) Enlarged view of LSV from −0.18 to −0.12 V, exhibiting the rate of H_2_ evolution. (**d**) CV of Zn//Ti cells at 1 mV s^−1^. (**e**) Initial NOP tested at 1 mA cm^−2^ for Zn//Ti cells for 1 h of Zn plating. (**f**) CA of Zn anodes using a three-electrode system with a fixed overpotential of –150 mV using REF and 0.5% PEG200 + 0.2% QKL.

The enhanced cycling stability of the Zn anode is attributed to the reduced water-induced side reactions and guided Zn deposition, as analyzed in Figure 3. To determine the corrosion behavior on the Zn plate, the Tafel method was employed using a three-electrode system at a scan rate of 0.1 mV s^−1^. Table S4 (Supporting Information) presents the resulting Tafel curves with/without additives. The surface corrosion rates in 0.5% PEG200 + 0.2% QKL were found to be reduced from 5.368 for REF to 0.198 mA cm^−2^ with the increase in the potential for the corrosion reaction from −0.999 to −0.909 V, as displayed in Figure 3a. For comparison, Figure S4 (Supporting Information) demonstrates that QKL, along with PEG200, mixed into the aqueous electrolyte contributed to the increasing corrosion potential, which was much higher than 0.5% PEG200, indicating the probability of the cationic quaternary ammonium groups’ interference with the Zn^2+^ solvation structure during the de-solvation process. As a result, not only can Zn sulfate byproduct formation be mitigated by reducing water activity on the Zn surface, but this can also reduce the energy barrier for the de-solvation process [41]. Later, the potential for HER was measured by LSV on the Zn//Ti cells at a scan rate of 1 mV s^−1^, as shown in Figure 3b. As the potential was increased from −0.075 V for REF to −0.132 V, the 0.5% PEG200 + 0.2% QKL in ZnSO_4_ suppressed H_2_ evolution, protecting the Zn anode from deterioration caused by the loss of inherited electrons of the metal [42]. As revealed in Figure 3c, the rate of H_2_ evolution further plummeted to −1.233 mA cm^−2^ at −0.15 V, indicating the retardation of HER. Thus, with the lower dosage of QKL combined with 0.5% PEG200, the water-side reactions can be inhibited, and this results in improving the electrochemical performance of the Zn anode in the aqueous environment.

For the deposition behavior, CV was performed on the Zn//Ti cells to determine the NOP, whereby divalent Zn cations are reduced and plated on the substrate. A strong nucleation driving force and a small grain of the nuclei can be observed when a high NOP value is applied, which suggests compact and uniform Zn deposition [43]. In our case, the NOP in 0.5% PEG200 + 0.2% QKL was much improved, by 53 mV, compared to REF, as shown in Figure 3d, implying smooth and dendrite-free deposition. This improved deposition can be attributed to the mutual adsorption effects on the anode, as observed in Figure S5 (Supporting Information), showing increases in NOP in 0.5% PEG200, 0.5% PEG200 + 0.1% QKL, and 0.5% PEG200 + 0.5% QKL. Consistently, an enhanced initial NOP was observed when the Zn//Ti cells were tested at 1 mA cm^−2^, resulting in a higher value of 82.3 mV for 0.5% PEG200 + 0.2% QKL as compared to 43 mV for REF, as shown in Figure 3e. The small nuclei formation seen for Zn particles is supported using the co-additive electrolyte system and means that dendrite-free deposition can be achieved, extending the reversibility and cycling stability of the Zn anode. Consequently, 0.5% PEG200 + 0.2% QKL showed steady current density over 1000 s after the initial nucleation process, after which a rapid decrease in current density could be observed, as shown in Figure 3f. In comparison, the current density continuously increased over time for REF, suggesting the occurrence of two-dimensional (2D) deposition, which may help to form dendrites on the surface. The constant lower current density indicates that Zn deposition is homogenous, which, in our case, is favored by the capacity of PEG200 and QKL to absorb onto the anode, distributing the Zn^2+^ ions flux uniformly during the plating process.

### 3.4. Stability and Reversibility of the Zn Anode

**Figure 4:**
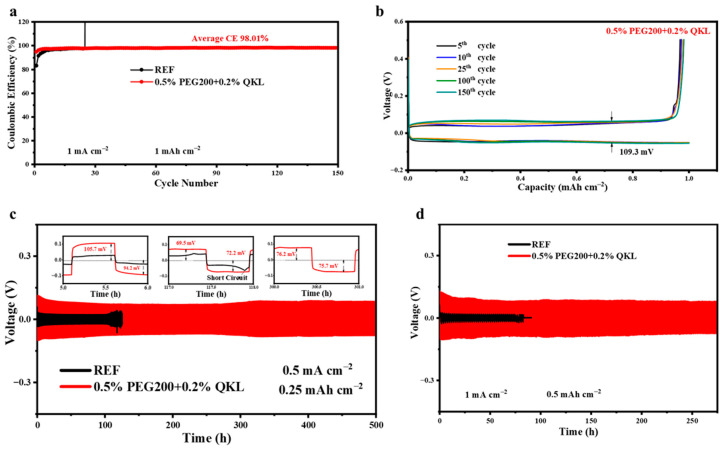
Zn anode reversibility and cycling stability in REF and 0.5% PEG200 + 0.2% QKL. (**a**) Coulombic efficiency (CE) of Zn//Cu asymmetric cells. (**b**) Voltage–capacity profile for 0.5% PEG200 + 0.2% QKL. (**c**) Cycling performance of Zn//Zn symmetric cells at 0.5 mA cm^−2^, with the enlarged view representing voltage hysteresis during the stripping/plating process. (**d**) Zn//Zn symmetric cells at 1 mA cm^−2^ and 0.5 mAh cm^−2^.

To identify the optimized co-additive, the CE on the Zn//Cu cells was tested with/without additives. The optimal performance was demonstrated by 0.5% PEG200 + 0.2% QKL, maintaining 98.01% CE for 150 cycles, whereas the REF battery suffered a short circuit before 30 cycles at 1 mA cm^−2^ and 1 mAh cm^−2^, as revealed in Figure 4a. Meanwhile, 0.5% PEG200, 0.5% PEG200 + 0.1% QKL, and 0.5% PEG200 + 0.5% QKL showed insignificant improvements in stability, as shown in Figure S6a (Supporting Information). Moreover, the voltage–capacity profile for the optimized co-additive demonstrates improved reversibility as compared to REF, with higher overpotential, as exhibited in Figure 4b and Figure S6b (Supporting Information), respectively.

Afterward, the Zn//Zn symmetric cells demonstrated a highly satisfactory performance when we utilized 0.5% PEG200 + 0.2% QKL, as compared to REF at 0.5 mA cm^−2^ (Figure 4c) and 1 mA cm^−2^ (Figure 4d), maintaining stability for 500 and around 280 h, respectively. On the contrary, the symmetric cells experienced a short circuit before 130 h at 0.5 mA cm^−2^ and around 90 h at 1 mA cm^−2^ when tested in 2 M ZnSO_4_ + 0.2 M MnSO_4_. As revealed in Figure S7a,b (Supporting Information), other co-additives, such as 0.5% PEG200 + 0.1% QKL and 0.5% PEG200 + 0.5% QKL, also enhanced Zn reversibility and cycling stability; however, the batteries showed less of an improvement as compared to 0.5% PEG200 + 0.2% QKL. Thus, the reversibility and cycling stability of the Zn anode can be enhanced with uniform Zn deposition by adding optimized dosages of PEG200 and QKL into the ZnSO_4_-based aqueous electrolyte. During the stripping/plating process with a 0.5 mAh cm^−2^ current density and a 0.25 mAh cm^−2^ areal specific capacity, the voltage hysteresis in 0.5% PEG200 + 0.2% QKL reached a much higher value than in REF in its initial cycles, as displayed in the insets in Figure 4c. In contrast, because of the combined effects of PEG200 and QKL molecules’ adsorption on the Zn anode’s surface, which can guide Zn^2+^ flux evenly, the voltage hysteresis was shown to reduce afterward, and the difference between plating (76.2 mV) and stripping (75.7 mV) at the 300th cycling step reached only 0.5 mV. These results prove that 0.5% PEG200 + 0.2% QKL is the optimal co-additive for use in Zn sulfate-based electrolytes to improve Zn reversibility over long periods of cycling.

### 3.5. Zn//MnO_2_ Battery Performance

**Figure 5:**
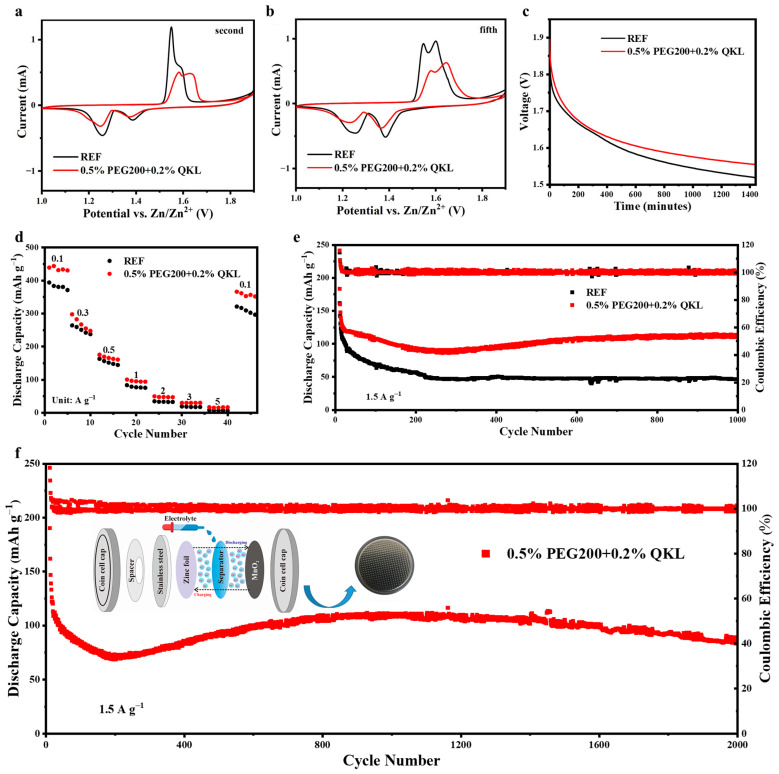
Zn//MnO_2_ full coin cell performance in REF and 0.5% PEG200 + 0.2% QKL. CV of the Zn//MnO_2_ battery at the (**a**) 2nd scan and (**b**) 5th scan. (**c**) Open circuit voltage (OCV) profile of batteries. (**d**) Rate performance from 0.1 to 5 A g^−1^ for full batteries. (**e**) Cycling performance of full cells Zn//MnO_2_ in REF and 0.5% PEG200 + 0.2% QKL at a current density of 1.5 A g^−1^ for 1000 cycles and (**f**) long-term cycling for 2000 cycles using the MnO_2_ cathode in 0.5% PEG200 + 0.2% QKL.

CV was performed at a scan rate of 0.1 mV s^−1^ on the Zn//MnO_2_ full cells over one to five scans using REF and electrolyte additives, as displayed in Figure S8 (Supporting Information). During the first scan, a single cathodic peak at ~1.22 V could be observed, while a corresponding anode peak appeared at 1.55 V with/without additives. In the following cycles (from the second to the fifth), new cathode peaks arose at ~1.38 and 1.25 V, and, in the meantime, the anode peak split into two coupled peaks at 1.55 and 1.66 V, corresponding to a two-step Zn insertion and extraction mechanism [31,44,45]. Compared to REF, the strength of the redox peaks for 0.5% PEG200 + 0.2% QKL appeared to be reduced in the second and fifth scans, as displayed in Figure 5a,b. However, the two reversible redox peaks in the cathode and anodic steps were obvious, indicating there was no significant effect on the energy storage mechanism when utilizing an optimized co-additive. To analyze the weakening strength of redox peaks in 0.5% PEG200 + 0.2% QKL, the EIS was recorded from 0.1 to 10^5^ Hz on the full cells, as depicted in Figure S9 (Supporting Information). The batteries demonstrated a semi-circular behavior and showed a higher charge transfer resistance for the optimized co-additive as compared to REF. Thus, 0.5% PEG200 + 0.2% QKL contributed to slightly higher impedance, as presented in Table S5 (Supporting Information), which is reflected in the weakening redox peaks of CV profiles. Later on, the Zn//MnO_2_ batteries were tested under the OCV condition to identify the effect of the co-additive on reducing self-discharge during resting hours. As shown in Figure 5c, 0.5% PEG200 + 0.2% QKL demonstrated an inhibitory effect on the self-discharge of batteries with higher remaining potential after 24 h than REF. In addition, the optimized co-additive resulted in the slightly improved rate performance of the full cells with higher specific capacities under the same current densities, as observed in Figure 5d.

Afterward, the cycling stability of the Zn//MnO_2_ batteries was examined at a current density of 1.5 A g^−1^ with/without additives. Considering the effectiveness of 0.5% PEG200 + 0.2% QKL in reducing water-induced reactions and dendritic growth, the batteries retained a decent specific capacity of above 110 mAh g^−1^ after 1000 cycles, whereas REF delivered less than 50 mAh g^−1^, as demonstrated in Figure 5e. Furthermore, Figure S10 (Supporting Information) shows the charge/discharge profiles, revealing an improved energy storage capacity for the co-additive as compared to REF during cycling. In comparison, Figure S11 (Supporting Information) represents the cycling performances of other additives, showing less stability with lower capacity retention. Therefore, the long-term cyclability was further tested for 0.5% PEG200 + 0.2% QKL at 1.5 A g^−1^, as shown in Figure 5f, and this resulted in a satisfactory capacity retention of ~85 mAh g^−1^ after 2000 cycles. Table S6 compares our work, in terms of the cyclability of the Zn//MnO_2_ full battery using electrolyte additives, with other reported works, demonstrating the significant contribution our optimized electrolyte makes to improving battery life.

### 3.6. Mechanism of Protection on the Zn Anode by Electrolyte Co-Additive

**Figure 6:**
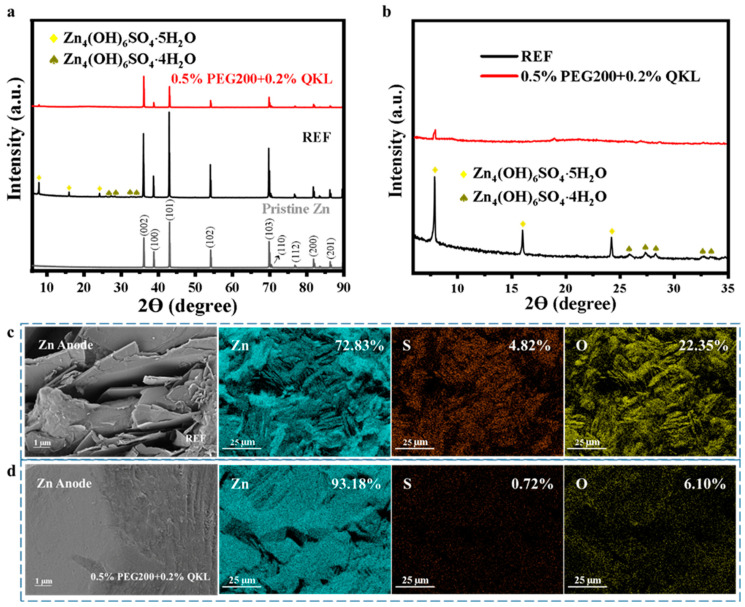
Characterization of the Zn anode after 1000 cycles in full battery Zn//MnO2 at 1.5 A g^−1^. (**a**) XRD of the anode in REF and 0.5% PEG200 + 0.2% QKL. (**b**) Enlarged view of XRD from 6 to 35 2θ (degrees). (**c**,**d**) SEMs of the cycled anode in REF and 0.5% PEG200 + 0.2% Q KL with EDS mapping, respectively.

To investigate the crystal structure, the XRD results of the pristine and cycled Zn anode after 1000 cycles of testing in the full batteries are shown in Figure 6a. Unlike REF, with 101 as the dominant peak, the 002-plane appeared to show the highest peak intensity when using 0.5% PEG200 + 0.2% QKL. This predominant (002) texture is thought to be advantageous since it has been shown that parallel grain alignment is more resilient to detrimental reactions and dendritic development [46,47,48,49]. Thus, an electrolyte containing 0.5% PEG200 + 0.2% QKL as a co-additive gives rise to a horizontal arrangement of Zn deposition. It has been revealed that the selective adsorption of PEG onto particular crystallographic faces of the developing nuclei is responsible for the (002) texture [50]. This behavior will selectively restrict Zn^2+^ ions’ access to selected crystal planes, inducing the preferred orientation. Figure 6b displays an enlarged view of XRD, with a specific range of 2θ representing corrosion-induced byproduct peaks in REF and 0.5% PEG200 + 0.2% QKL. The ZHS byproduct is formed by complexation between Zn^2+^/SO_4_^2−^ and OH^−^, which takes place in the electrolyte during the H^+^-induced corrosion of the Zn anode and the subsequent release of H_2_ from the electrolyte [51,52,53]. ZHS is reduced by a significant amount after employing the optimized co-additive, as it can be observed that the intensity of the byproduct-related peaks here is weaker than in REF. This can be attributed to the combined effects of PEG200 and QKL, together reducing the corrosion rate and increasing the potential for H_2_ evolution, as revealed by the Tafel plot and LSV curves.

These results align with the SEM images and EDS results, revealing Zn, S, and O elemental mapping, as observed in Figure 6c,d, and showing a cycled Zn anode morphology in REF and 0.5% PEG200 + 0.2% QKL. When comparing this with pristine Zn with a smooth surface, as shown in Figure S12 (Supporting Information), the surface texture was found to be deteriorated, with unregulated and coarse deposition and an accumulation of ZHS formation, as depicted by the vertically oriented Zn deposits that followed cycling in REF with the consumed (dead) Zn anode. The S and O contents increased significantly for REF compared to pristine Zn, as revealed by the EDS mapping. However, after using 0.5% PEG200 + 0.2% QKL, smooth dendrite-free deposition was observed on the Zn anode, and the EDS mapping further confirmed the significantly reduced S and O elemental contents, indicating the mitigation of ZHS accumulation on the cycled Zn anode’s surface.

**Figure 7:**
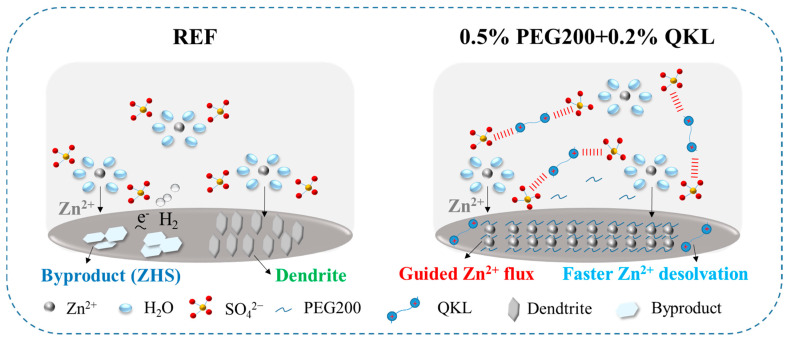
Schematic illustration of Zn deposition behavior in REF and 0.5% PEG200 + 0.2% QKL.

Figure 7 illustrates the effects of REF and 0.5% PEG200 + 0.2% QKL electrolytes on the Zn deposition behavior. PEG200 shows strong absorbability on the Zn anode due to the presence of abundant hydroxyl (–OH) groups and the ability to support uniform Zn cation flux on the 002-plane across the surface, enabling dendrite-free deposition. Meanwhile, QKL contributes to improving Zn nucleation sites and ensures the faster de-solvation of Zn cations due to the effect on the solvation structure. This weakening interaction between water molecules and Zn^2+^ cations can lower the de-solvation energy barrier during the Zn deposition process [54,55]. Subsequently, benefitting from PEG200 and QKL’s higher affinity for the Zn anode and the bonding between the electrophilic group (CH_3_)_3_N^+^ and SO_4_^2-^, homogenous Zn deposition is exhibited, with unnoticeable dendritic formation and reduced byproducts, when using the optimized co-additive electrolyte. On the contrary, the Zn anode in REF exhibited dendrite and ZHS formation, which are attributed to the uncontrolled Zn deposition and the interrelated corrosion and HER. Therefore, 0.5% PEG200 + 0.2% QKL, when used as a co-additive electrolyte, plays a distinct role in suppressing the formation of Zn dendrites and inhibiting water-side reactions on the surface of the Zn electrode.

## 4. Conclusions

In conclusion, we designed a safe, inexpensive, and nontoxic co-additive for Zn//MnO_2_ batteries via a facile strategy using compound additives. After utilizing 0.5% PEG200 + 0.2% QKL in 2 M ZnSO_4_ + 0.2 M MnSO_4_, the Zn//Zn symmetric cells displayed improved cycling stability (500 h at 0.5 and 280 h at 1 mA cm^−2^). The homogenous Zn deposition and reduced water-induced reactions can be ascribed to the synergistic effects of PEG200 and QKL, as affirmed by corrosion, LSV, CE, CA, and NOP electrochemical tests. Benefitting from the high affinity of PEG200 towards the Zn anode and the regulating effect of QKL on the Zn coordination environment, coin cell assembled Zn//MnO_2_ batteries exhibited a higher discharge capacity of around 110 mAh g^−1^ for 1000 cycles and 85 mAh g^−1^ after 2000 cycles at 1.5 A g^−1^. Furthermore, XRD and SEM confirmed the smooth morphology of the Zn anode surface dominated by (002) textured deposition in 0.5% PEG200 + 0.2% QKL. With notable effectiveness in improving performance, here, we presented an approach using combined additives that does not compromise on the low-cost and eco-friendly goals of electrolyte additives.

## Data Availability

The data presented in this study are available on request from the corresponding author.

## Supplementary Materials

The following supporting information can be downloaded at https://www.mdpi.com/article/10.3390/polym17070888/s1. Figure S1: Diagram of coin cells. (a) Zn//MnO_2_ batteries; (b) Zn//Zn symmetric cells. Figure S2: ^1^H NMR for (a) KL and (b) QKL. Figure S3: (a) FTIR of electrolytes corresponding to the peak representation of functional groups with/without additives. (b) v-SO_4_^2−^ group for REF, 0.5% PEG200, 0.5% PEG200 + 0.1% QKL, and 0.5% PEG200 + 0.5% QKL. Figure S4: Tafel curves showing the corrosion behavior of other electrolyte additives. Figure S5: NOP on Zn//Ti cells at a scan rate of 1 mV s^−1^ in REF, 0.5% PEG200, and 0.5% QKL. Figure S6: (a) Coulombic efficiency (CE) of control (0.5% PEG200) and co-additives (0.5% PEG200 + 0.1% QKL and 0.5% PEG200 + 0.5% QKL). (b) Voltage–capacity profiles for REF. Figure S7: Cyclic performance of Zn//Zn symmetric cells for 0.5% PEG200, 0.5% PEG200 + 0.1% QKL, and 0.5% PEG200 + 0.5% QKL at current densities of (a) 0.5 mA cm^−2^ and (b) 1 mA cm^−2^. Figure S8: CV profiles of Zn//MnO_2_ tested at 0.1 mV s^−1^ with/without additives. Figure S9: Electrochemical impedance spectroscopy (EIS) on Zn//MnO_2_. Figure S10: Charge/discharge profiles for REF and 0.5% PEG200 + 0.2% QKL. Figure S11: Cycling performance of Zn//MnO_2_ for 1000 cycles at 1.5 A g^−1^ using 0.5% PEG200, 0.5% PEG200 + 0.1% QKL, and 0.5% PEG200 + 0.5% QKL. Figure S12: SEM image of pristine Zn with EDS mapping. Table S1: Zeta potential values for KL and QKL at pH 4.5. Table S2: Elemental composition of KL and QKL. Table S3: Ionic conductivities of electrolyte samples. Table S4: Measured values of the corrosion rate derived from Tafel curves for REF and electrolyte with co-additives. Table S5: Fitted data using an equivalent circuit for the EIS of full batteries. Table S6: Comparison of our work on Zn//MnO_2_ batteries with the published literature after applying electrolyte additives [56,57,58,59,60].
