# Supplementary material for: Improved Performances of Zn//MnO2 Batteries with an Electrolyte Containing Co-Additives of Polyethylene Glycol and Lignin Derivatives
Source: Polymers (Basel). 2025 Mar 26;17(7):888. doi: 10.3390/polym17070888 (PMC11991002; doi:10.3390/polym17070888)
Supplement: Supplementary file 1 [file polymers-17-00888-s001.zip › polymers-3515425-supplementary.pdf]

Supporting Information

Improved performances of Zn//MnO_2 batteries with an electrolyte containing co-additives of polyethylene glycol and lignin derivatives

Muzammil Hussain Memon[1,2], Md. Asraful Alam[1,2], Qiyuan Xie[1,2], Abdul Rahman Abbasi[1,2], Lele Wang[1,2], Jingliang Xu[1,2,3]*, Wenlong Xiong[1,2,3,]*

[1] State Key Laboratory of Cotton Bio-breeding and Integrated Utilization, School of Chemical Engineering, Zhengzhou University, Zhengzhou 450001, China

[2] State Key Laboratory of Biobased Transportation Fuel Technology, School of Chemical Engineering, Zhengzhou University, Zhengzhou 450001, China

[3] Henan Center for Outstanding Overseas Scientists, Zhengzhou University, Zhengzhou 450001, China

* Correspondence: xujl@zzu.edu.cn (J. Xu); xiongwenlong@zzu.edu.cn (W. Xiong)

Academic Editor: Firstname Lastname

Received: date
Revised: date
Accepted: date
Published: date

Citation: To be added by editorial staff during production.

Copyright: © 2025 by the authors. Submitted for possible open access publication under the terms and conditions of the Creative Commons Attribution (CC BY) license (https://creativecommons.org/licenses/by/4.0/).

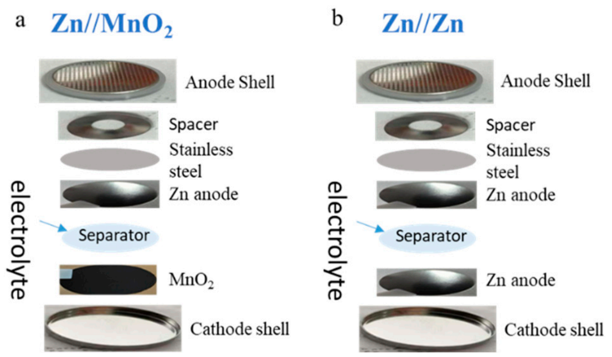

Figure S1. Diagram of coin-cells (a) $Zn//MnO_2$ full batteries, (b) Zn//Zn symmetric cells.

Table S1. Zeta potential values for KL and QKL at pH 4.5.

Experiment No.	Zeta potential (mV)	
	KL	QKL
1.	–36.058	24.063
2.	–37.978	23.525
3.	–36.093	22.012
Avg.	–36.709	23.2

Table S2. Elemental composition of KL and QKL

**Samples**	**Elemental content（%）**			
	**C**	**H**	**N**	**S**
KL	62.426	6.094	0.127	1.759
QKL	56.085	6.774	2.387	1.972

Table S3. Ionic conductivities of electrolyte samples

**Electrolyte Samples**	**Ionic conductivity (mS cm^{-1})**
2 M $ZnSO_4$ + 0.2 M $MnSO_4$ (REF)	**43**
0.5% PEG200	**41**
0.5% PEG200+0.1% QKL	**40**
0.5% PEG200+0.2% QKL	**40**
0.5% PEG200+0.5% QKL	**39**

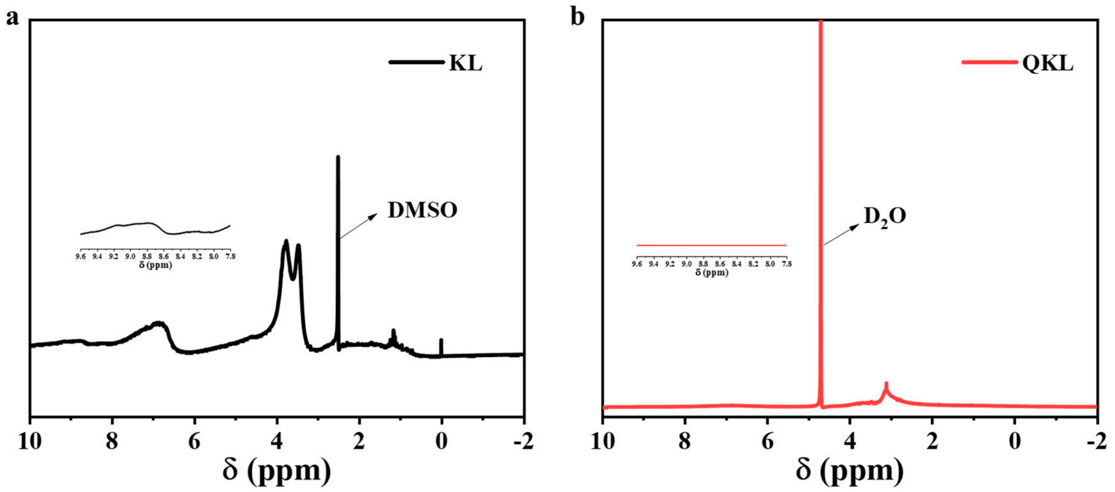

Figure S2. ^{1}H NMR for (a) KL and (b) QKL.

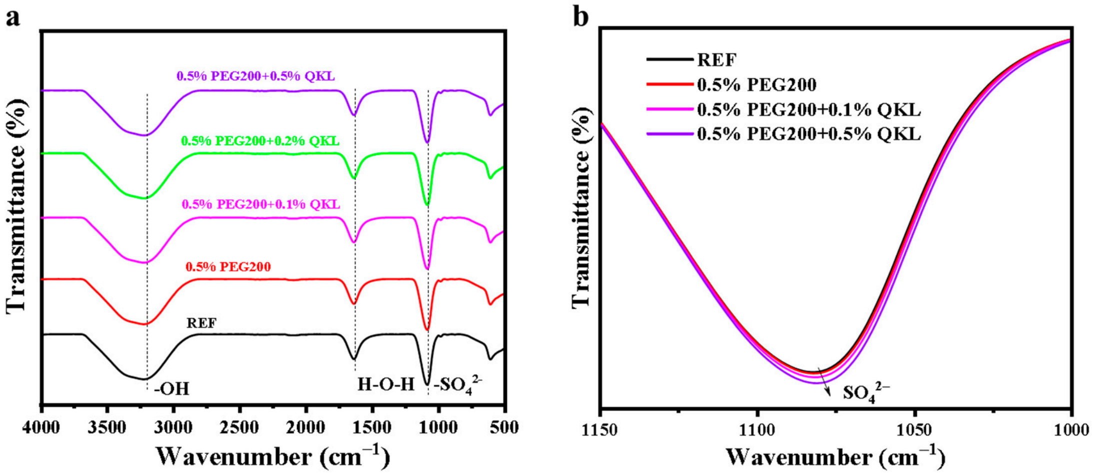

Figure S3. (a) FTIR of electrolytes corresponding with peak representation of functional groups with/without additives. (b) v-SO_4^{2-} group for REF, 0.5% PEG200, 0.5% PEG200+0.1% QKL and 0.5% PEG200+0.5% QKL.

Table S4. Measured values of corrosion rate derived from Tafel-curves for REF and electrolyte with co-additives.

Electrolyte	Corrosion potential (V)	Corrosion current (mA)	Contact area (cm^2)	Corrosion current density (mA cm^{-2})
**REF**	-0.999	2.684	0.5	5.368
**0.5% PEG200**	-0.998	0.6588	0.4	1.647
**0.5% PEG200+0.1% QKL**	-0.955	0.0713	0.35	0.203
**0.5% PEG200+0.2% QKL**	-0.909	0.1092	0.55	0.198
**0.5% PEG200+0.5% QKL**	-0.913	0.0639	0.3	0.213

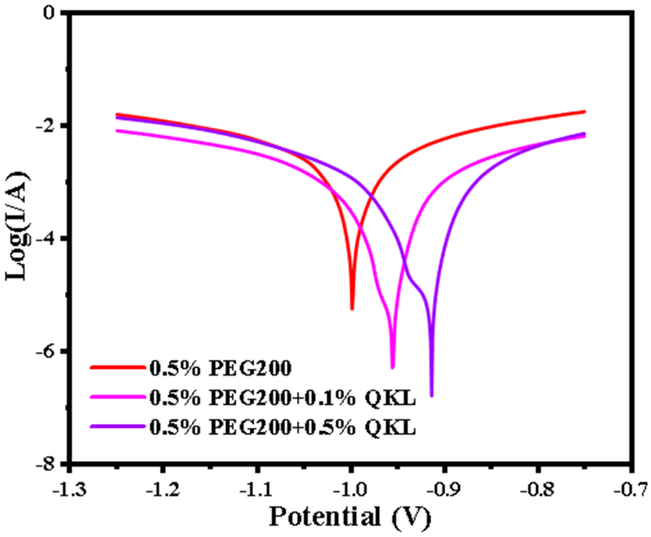

Figure S4. Tafel curves showing corrosion behavior of other electrolyte additives.

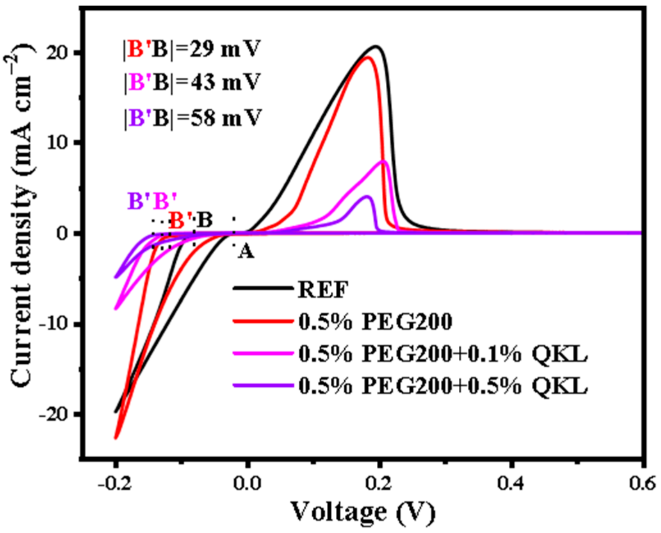

Figure **S5.** NOP on Zn//Ti cells at scan rate of 1 mV s^{-1} in REF, 0.5% PEG200 and 0.5% QKL.

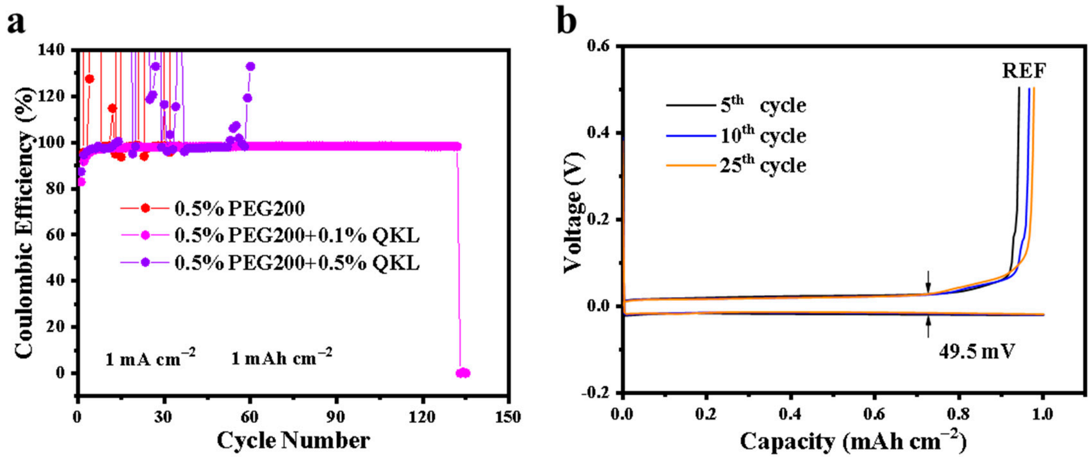

Figure S6. (a) Coulombic efficiency (CE) of control (0.5% PEG200) and co-additives (0.5% PEG200 + 0.1% QKL and 0.5% PEG200 + 0.5 % QKL). (b) Voltage-capacity profiles for REF.

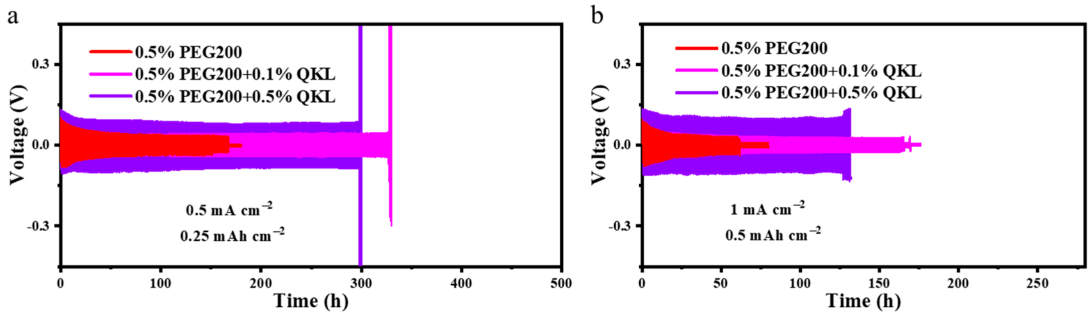

Figure S7. Cyclic performance of Zn//Zn symmetric cells for 0.5% PEG200, 0.5% PEG200 + 0.1% QKL and 0.5% PEG200 + 0.5% QKL at current densities of (a) 0.5 mA cm^{-2} and (b) 1 mA cm^{-2}.

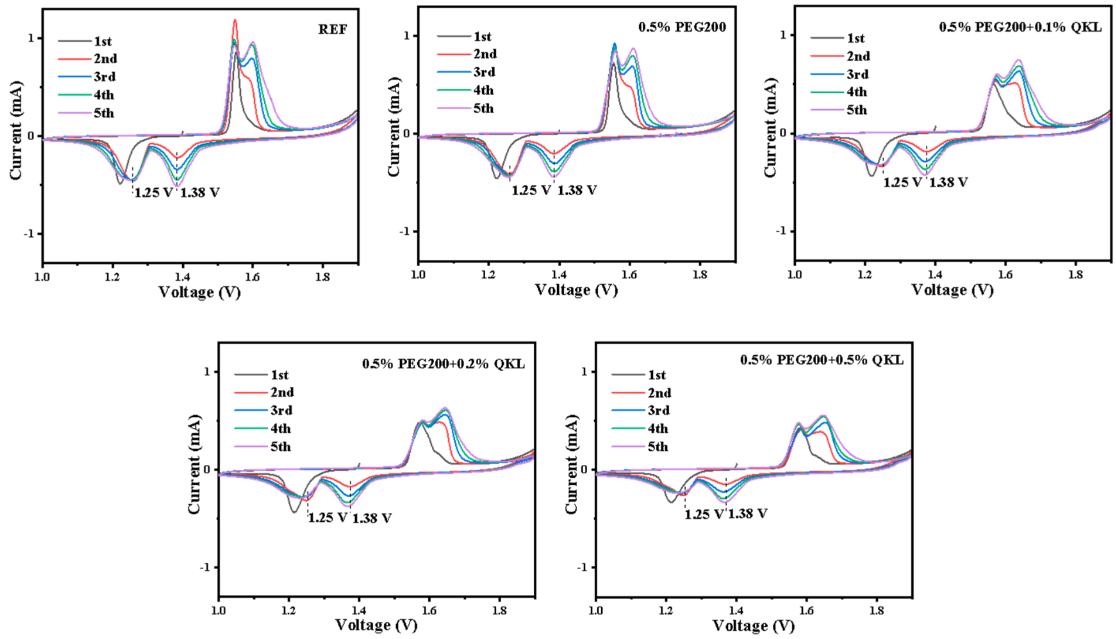

Figure **S8.** CV profiles of Zn//MnO_2 tested at 0.1 mV s^{-1} with/without additives.

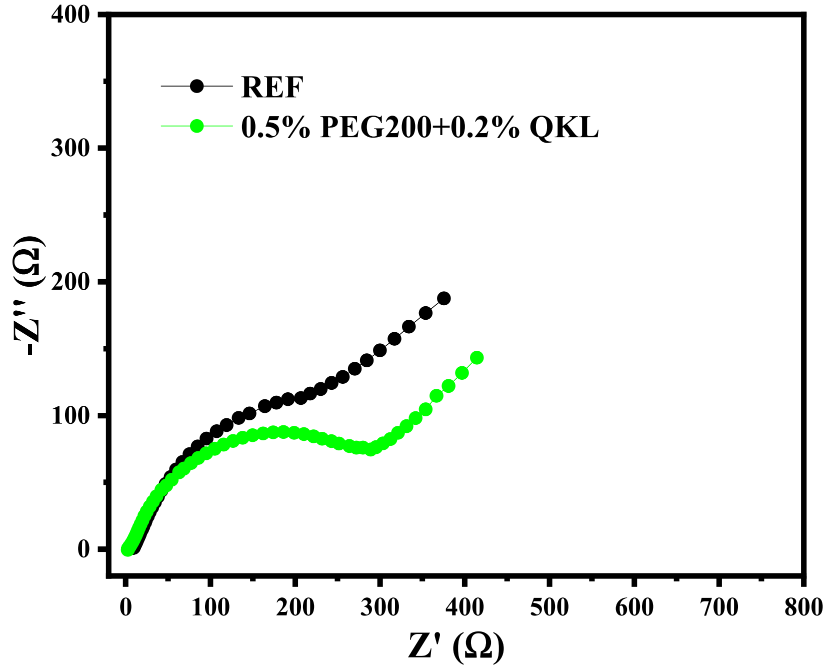

Figure S9. Electrochemical impedance spectroscopy (EIS) on Zn//MnO_2.

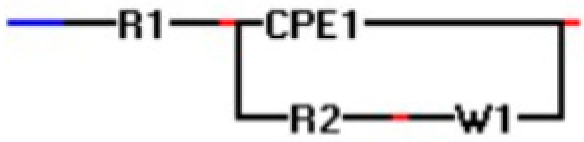

Table S5. Fitted-data using an equivalent circuit for the EIS of full batteries.

	**Solution resistance (R_s)**	**Charge transfer resistance (R_{ct})**	**Total resistance (R_t)**
**REF**	8.127	201.2	**209.2 Ω**
**0.5% PEG200 + 0.2% QKL**	2.443	267.7	**270.1 Ω**

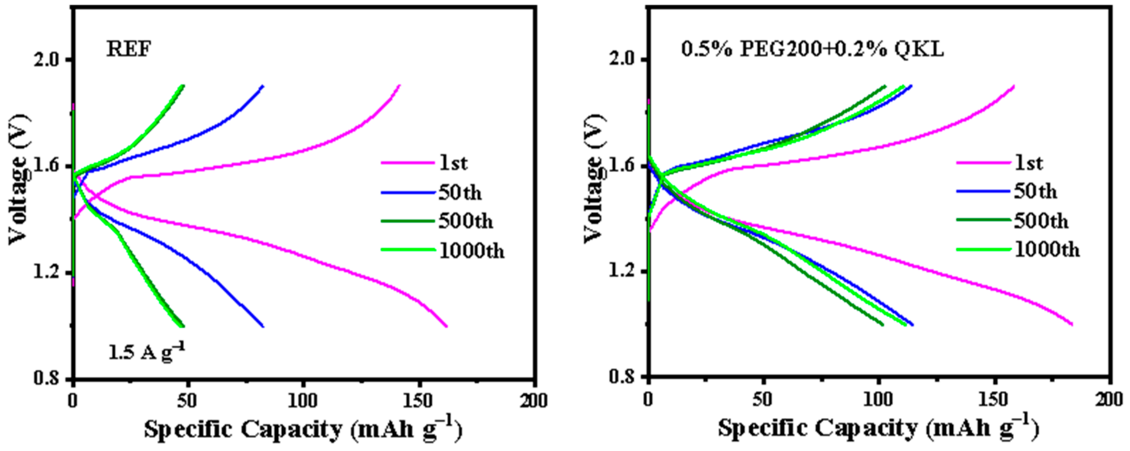

Figure **S10.** Charge/discharge profiles for REF and 0.5% PEG200 + 0.2% QKL

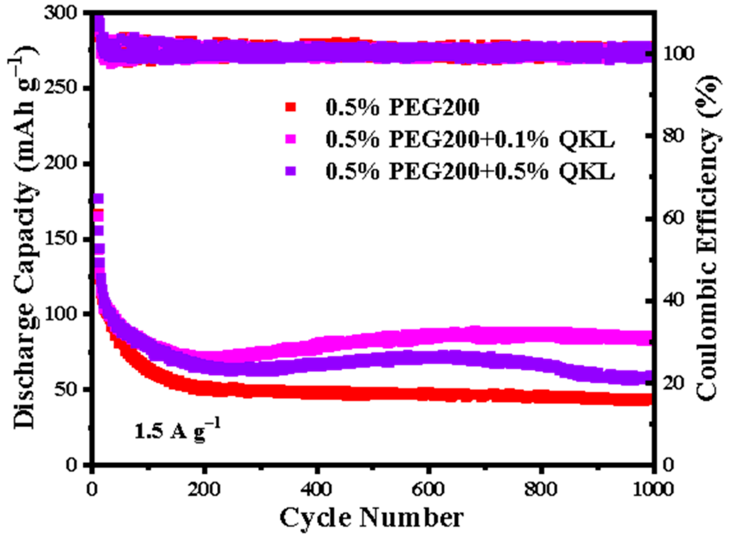

Figure **S11.** Cycling performance of Zn//MnO_2 for 1000 cycles at 1.5 A g^{-1} using 0.5% PEG200, 0.5% PEG200 + 0.1% QKL and 0.5% PEG200 + 0.5% QKL

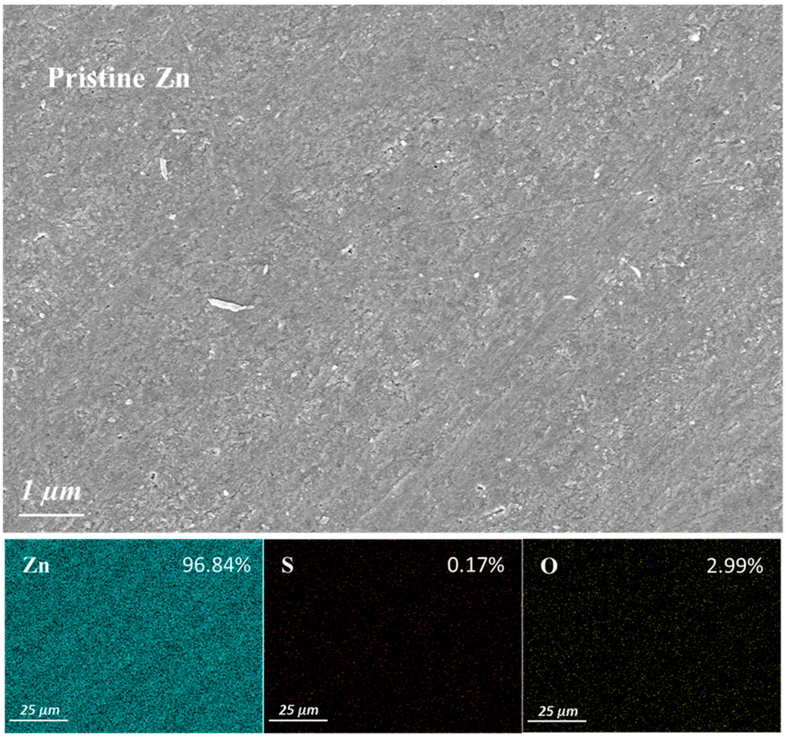

Figure S12. SEM image for Pristine Zn with EDS mapping.

Table S6 Comparison of our work on Zn//MnO_2 batteries with published literature after using electrolyte additives.

Electrolyte + Additives	Cathode	Current density ($A g^{-1}$)	Specific capacity ($mAh g^{-1}$)	Cycle number	Ref.
$ZnSO_4$ + Polypropylene glycol	MnO_2	1	~100	300	[1]
$ZnSO_4$ + Penta-potassium triphosphate	MnO_2	1	< 90	300	[2]
$ZnSO_4$ + Ethyldiamine tetraacetic acid disodium salt	MnO_2	1	~ 85	200	[3]
$ZnSO_4$ + glutamic acid	MnO_2	1	< 100	300	[4]
1 M $ZnSO_4$ + 0.5 M Na_2SO_4 + PAM	MnO_2	1	< 100	600	[5]
**2 M $ZnSO_4$ + 0.2 M $MnSO_4$ + (0.5% PEG200 + 0.2% QKL)**	**MnO_2**	**1.5**	**110**	**1000**	**This work**
			**85**	**2000**	

References

1. Li, J.; Zhou, S.; Chen, Y.; Meng, X.; Azizi, A.; He, Q.; Li, H.; Chen, L.; Han, C.; Pan, A. Self-Smoothing Deposition Behavior Enabled by Beneficial Potential Compensating for Highly Reversible Zn-Metal Anodes. *Advanced Functional Materials* **2023**, *33*, 2307201, https://doi.org/10.1002/adfm.202307201.
2. Yu, Y.; Zhang, P.; Wang, W.; Liu, J. Tuning the Electrode/Electrolyte Interface Enabled by a Trifunctional Inorganic Oligomer Electrolyte Additive for Highly Stable and High-Rate Zn Anodes. *Small Methods* **2023**, *7*, 2300546, https://doi.org/10.1002/smtd.202300546.
3. Cao, J.; Zhang, D.; Chanajaree, R.; Yue, Y.; Zeng, Z.; Zhang, X.; Qin, J. Stabilizing zinc anode via a chelation and desolvation electrolyte additive. *Advanced Powder Materials* **2022**, *1*, 100007, https://doi.org/10.1016/j.apmate.2021.09.007.
4. Li, C.; Gou, Q.; Tang, R.; Deng, J.; Wang, K.; Luo, H.; Cui, J.; Geng, Y.; Xiao, J.; Zheng, Y.; et al. Electrolyte Modulation of Biological Chelation Additives toward a Dendrite-Free Zn Metal Anode. *The Journal of Physical Chemistry Letters* **2023**, *14*, 9150-9158, https://doi.org/10.1021/acs.jpclett.3c02327.
5. Zhang, Q.; Luan, J.; Fu, L.; Wu, S.; Tang, Y.; Ji, X.; Wang, H. The Three-Dimensional Dendrite-Free Zinc Anode on a Copper Mesh with a Zinc-Oriented Polyacrylamide Electrolyte Additive. *Angewandte Chemie International Edition* **2019**, *58*, 15841-15847, https://doi.org/10.1002/anie.201907830.
